# Histopathological Evaluation of the Anterior Cruciate Ligament in Patients With Advanced Gonarthrosis

**DOI:** 10.1111/1756-185X.70160

**Published:** 2025-03-05

**Authors:** Elif Polat, Burak Gunaydin, Sevil Karabağ, Nurettin Heybeli

**Affiliations:** ^1^ Histology and Embryology Department Tekirdag Namik Kemal University Tekirdağ Turkey; ^2^ Orthopaedics and Traumatology Department Çorlu Optimed Hospital Tekirdağ Turkey; ^3^ Medical Pathology Department Tekirdag Namik Kemal University Tekirdağ Turkey; ^4^ Orthopaedics and Traumatology Department Tekirdag Namik Kemal University Tekirdağ Turkey

**Keywords:** anterior cruciate ligament, collagen, degeneration, gonarthrosis, histopathology

## Abstract

**Introduction:**

Knee osteoarthritis is a degenerative disease of the knee joint that leads to progressive loss of articular cartilage. There is only limited information in the literature on the degeneration of the anterior cruciate ligament (ACL) in patients with gonarthrosis. In this study, ACL samples excised during the surgery of patients undergoing total knee replacement (TKR) in 2019–2020 were evaluated histopathologically. The present study aims to investigate the relationship between the degeneration of the ACL and the stage of gonarthrosis.

**Methods:**

Direct X‐rays of 47 patients undergoing knee arthroplasty were evaluated and stratified into two groups: Stage 3 (Group 1) and Stage 4 (Group 2). ACL samples were examined histopathologically. Staining was performed with hematoxylin–eosin (H&E), Alcian blue, and Masson's trichrome. The degree of degeneration was determined according to the Movin score and Bonar score. Based on these scores, the statistical significance of the relationship between the stage of gonarthrosis and histopathological examination results was evaluated.

**Results:**

When Group 1 patients were graded according to total Movin scores, the result was mild degeneration in 72.7% of the patients, while the corresponding rates were 76% in Group 2. None of the patients had a score reflecting severe degeneration. The total Bonar scores showed mild degeneration in 68.2% of Group 1 patients. In Group 2, the scores showed mild degeneration in 52% of the patients. No statistically significant difference was found between the groups in either scoring.

**Conclusion:**

It was determined that comparable degeneration occurred in the ACL with the progression of the stage of gonarthrosis.

## 1 Introduction

Degenerative changes in the knee joint resulting from a variety of different reasons reduce the quality of life by causing pain and restriction of movement. Knee arthroplasty is a surgical treatment option that orthopedic surgeons commonly apply in patients with advanced stages (Stages 3–4) of degenerative osteoarthritis of the knee joint (gonarthrosis) [1].

ACL has a microstructure similar to that of other soft connective tissues [2]. This ligament consists of multiple fascicles, whose basic unit is collagen and is surrounded by connective tissue referred to as the paratenon. Histologically, three regions can be distinguished within the ACL. The proximal aspect is highly cellular, rich in round and oval cells, and contains some fusiform fibroblasts, collagen type II, and glycoproteins such as fibronectin and laminin. The middle aspect, containing fusiform and spindle‐shaped fibroblasts, is a special region of high‐density collagen fibers, cartilage, and fibrocartilage [3]. The distal aspect is rich in chondroblasts and oval fibroblasts and contains low‐density collagen bundles. The fibroblasts on both sides of these collagen bundles are round to oval in shape [4]. Two types of fibrils were defined by Strocchi and colleagues. The first type of fibrils is secreted by fibroblasts, has irregular outlines with variable diameters, and constitutes 50.3% of the entire ACL. The second type is a uniform type with regular margins, and these fibrils, secreted by fibro‐chondroblasts, constitute 43.7% of the ACL. These small homogeneous fibrils maintain the three‐dimensional organization of the ligament [5]. Cells and matrix components make up the remaining 6% of the ACL tissue [6].

Across different joint tissues, the articular cartilage appears to undergo both mechanical and age‐related degeneration [7]. The ACL is especially necessary for knee kinematics in rotational movements and also functions as a stabilizer in anterior and posterior gliding movements [8, 9]. The ACL, being the most commonly injured ligament of the knee, resists medial rotation, especially in the first 30° of knee flexion [10], as well as resisting varus and valgus strains.

In patients with gonarthrosis, conservative treatment methods such as activity restriction, weight loss, use of instruments such as crutches or a cane, anti‐inflammatory drugs, intra‐articular injections, and physical therapy are applied as the first‐line approach while various surgical treatment options are applied in cases where such conservative treatment fails [11]. Total knee arthroplasty is indicated in patients with advanced osteoarthritis who do not benefit from the aforementioned conservative treatment options [11, 12]. Arthroplasty is typically applied to patients aged 60 years and over with Stage 3 and Stage 4 gonarthrosis. In total knee arthroplasty operations, while the ACL is cut routinely, the posterior cruciate ligament may be cut or preserved depending on the prosthesis design [13].

The present study aims to investigate the presence of degeneration and evaluate the relationship between the stage of gonarthrosis and degeneration in the ligament using histopathological examination of ACL samples excised from patients undergoing total knee arthroplasty for gonarthrosis.

## 2 Methods

This study has been approved by the Local Ethics Committee as per the decision dated 02/22/2022 with the number 2020/143/06/05. All patients provided written informed consent before participating in the study. Radiographs taken during routine investigations of the 47 patients included in the study were evaluated, and these patients were stratified into two groups based on having Stage 3 or Stage 4 disease. Stage 3 patients constituted Group 1, and Stage 4 patients constituted Group 2. The team who performed the histological examinations performed these evaluations in a blinded fashion.

### 2.1 Inclusion Criteria

Patients over 50 years of age who have advanced gonarthrosis were included in the study.

### 2.2 Exclusion Criteria

Patients with chronic conditions other than hypertension, those without an anterior cruciate ligament, patients who had previously undergone surgical intervention (including arthroscopy) in the same knee, those with a history of infection in the same knee, patients with femoral or tibial fractures of the same extremity, and those with multiple ligament instability were not included in the study.

### 2.3 Radiological Evaluation

Knee X‐rays of the patients were evaluated and classified according to the Kellgren‐Lawrence classification system [14]. Joint narrowing, osteophytic lesions, subchondral cysts, and bone contour deformities were evaluated with this classification system [14].

### 2.4 Surgical Procedures and ACL Harvest

Preoperative antibiotic prophylaxis was administered to each patient. The patients were placed in the supine position, anesthesia was applied, and a tourniquet was placed in each case. After appropriate surgical site cleansing, sterile drapes were placed. A skin incision was made through the midline of the patella. The medial parapatellar approach, which is the standard procedure in total knee arthroplasty, was used for arthrotomy. After the patella was tilted laterally with the knee in extension, the joint space was exposed by positioning it in 90° flexion. At this point, the ACL was released from the femoral and tibial junctions with a scalpel and preserved as a sample for pathological examination. The subsequent stages of the surgery were applied in their respective order, and finally, the prosthesis was implanted.

### 2.5 Histopathological Examinations

**FIGURE 1:**
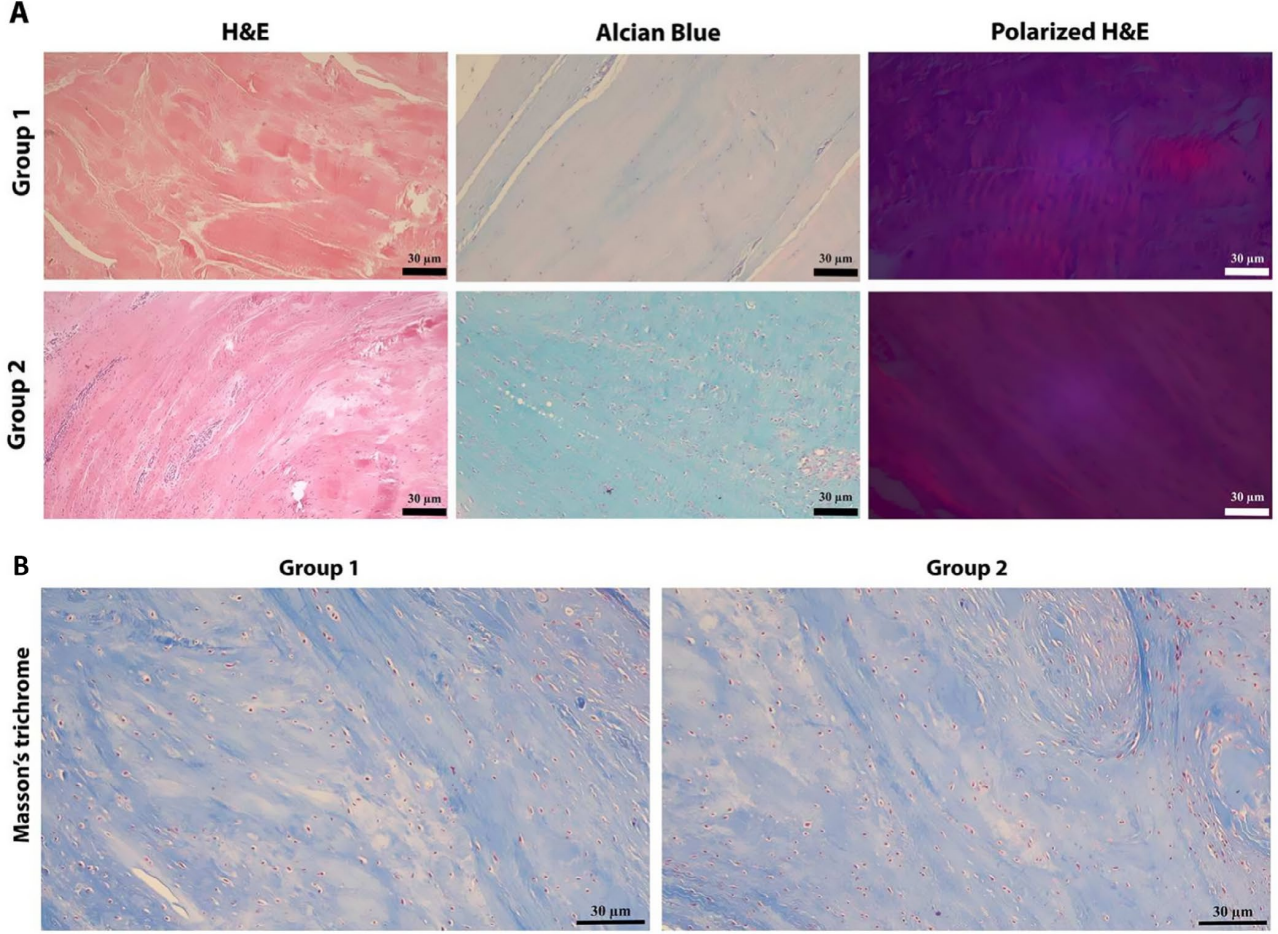
A Histopathological images of ACL (400×). B Masson's trichrome stain (400×).

Tissue sections were taken from the proximal part of the ACL. All the ACL samples were placed in 20 mL of sterile 10% formalin in a plastic container to be transferred to the pathology department. The samples were then dehydrated, embedded in paraffin, and cut to obtain 4‐μm sections. One section from each sample was routinely stained with hematoxylin and eosin (H&E) (Figure 1A). The two other sections were stained with Alcian blue (Figure 1A)(pH 2.5) (Rtu, Bio‐Optica, Milan, Italy) and Masson's trichrome (Figure 1B) (Rtu, Ventana, Tucson, AZ, USA) on the Ventana BenchMark Special Stains automated slide stainer (Roche Diagnostics, A.S, Istanbul, Turkey). The samples were stained with Masson's trichrome to highlight fibrosis [15] and with Alcian blue to highlight glycosaminoglycan (GAG) degeneration. All histopathological examinations were performed using an Olympus CX41 (Olympus, Japan) light microscope and an image analysis system (Kameram Gen III Image Analysis Software, Istanbul, Turkey). The samples stained with H&E were evaluated using both polarized and nonpolarized light microscopy.

Each slide was evaluated with a semi‐quantitative scale of Bonar and Movin scores modified by Maffulli as described elsewhere [16, 17]. The variables included in the Movin scale are (1) fiber structure; (2) fiber arrangement; (3) rounding of the nuclei; (4) regional variations in cellularity; (5) increased vascularity; (6) decreased collagen stainability; (7) hyalinization; and (8) GAG content [17]. Each variable was scored between 0 and 3, with 0 being normal, 1 slightly abnormal, 2 abnormal, and 3 markedly abnormal. The slides stained with H&E were used to evaluate the first seven variables, and those stained with Alcian blue were used to evaluate GAG content. The total semi‐quantitative histological score for a given slide could vary from 0 (normal tendon) to 24 (the most severe abnormality detectable) [17] (Figure 1A). The variables included in the Bonar scale are (1) cell morphology; (2) ground substance; (3) collagen; and (4) vascularity. In this scale, a 4‐point scoring system is used, where 0 indicates normal appearance and 3 indicates markedly abnormal appearance. Overall, the total score for a given slide may range from 0 (normal tendon) to 12 (the most severe abnormality detectable) [17] (Figure 1A).

For the evaluation of fibrosis, the thickness increase of the fibers was scored between 0 and 3 (Figure 1B).

### 2.6 Statistical Analyses

The SPSS 23.0 statistical package program (version 23.0; SPSS Inc., Chicago, IL, USA) was used to transfer the data obtained in this study to the computer environment and for statistical analyses. While evaluating the data, first, a test to check normal distribution was applied to all data. The data with normal distribution were presented as mean ± standard deviation (x ± SD). The data that were not normally distributed were presented as median values. An independent sample *t*‐test was applied to the data with normal distribution. The Mann–Whitney U test was used for the data that were not normally distributed. The Chi‐square test was used in the comparison of categorical variables. *p* < 0.05 was considered statistically significant.

## 3 Results

Twenty‐two of the 47 patients included in the study had Stage 3 disease and were allocated to Group 1, while 25 had Stage 4 disease and were allocated to Group 2. The mean age was 67.13 ± 6.79 years in Group 1 and 67.8 ± 6.75 years in Group 2. Group 1 consisted of 16 female and 6 male patients. Group 2 consisted of 18 female and 7 male patients. In Group 1, the affected knee was the right knee in 13 of the patients and the left knee in 9. In Group 2, the affected knee was the right knee in 14 of the patients and the left knee in 11 (Table 1).

**TABLE 1:** Demographics of patients with gonarthrosis by stage.

Stage		Group 1 (*n* = 22)	Group 2 (*n* = 25)	*p*
Age (year) (mean ± SD)		67.13 ± 6.79 (57–79)	67.8 ± 6.75 (52–77)	0.8
Gender	Female (%)	16 (47.1%)	18 (52.9%)	0.95
	Male (%)	6 (46.2%)	7 (53.8%)	
Side	Right (%)	13 (48.1%)	14 (51.9%)	0.83
	Left (%)	9 (45%)	11 (55%)	

The microscopic examination revealed mild degeneration in the fiber structure and fiber arrangements of patients with Stage 3 and Stage 4 disease. A mild‐to‐moderate loss of stability in collagen bundles was detected in polarized examinations. Mildly increased cellularity was observed homogeneously throughout the tissues. In the samples stained with Masson's trichrome, moderate fibrosis was detected in both groups. Alcian‐blue staining showed mild GAG degeneration.

**FIGURE 2:**
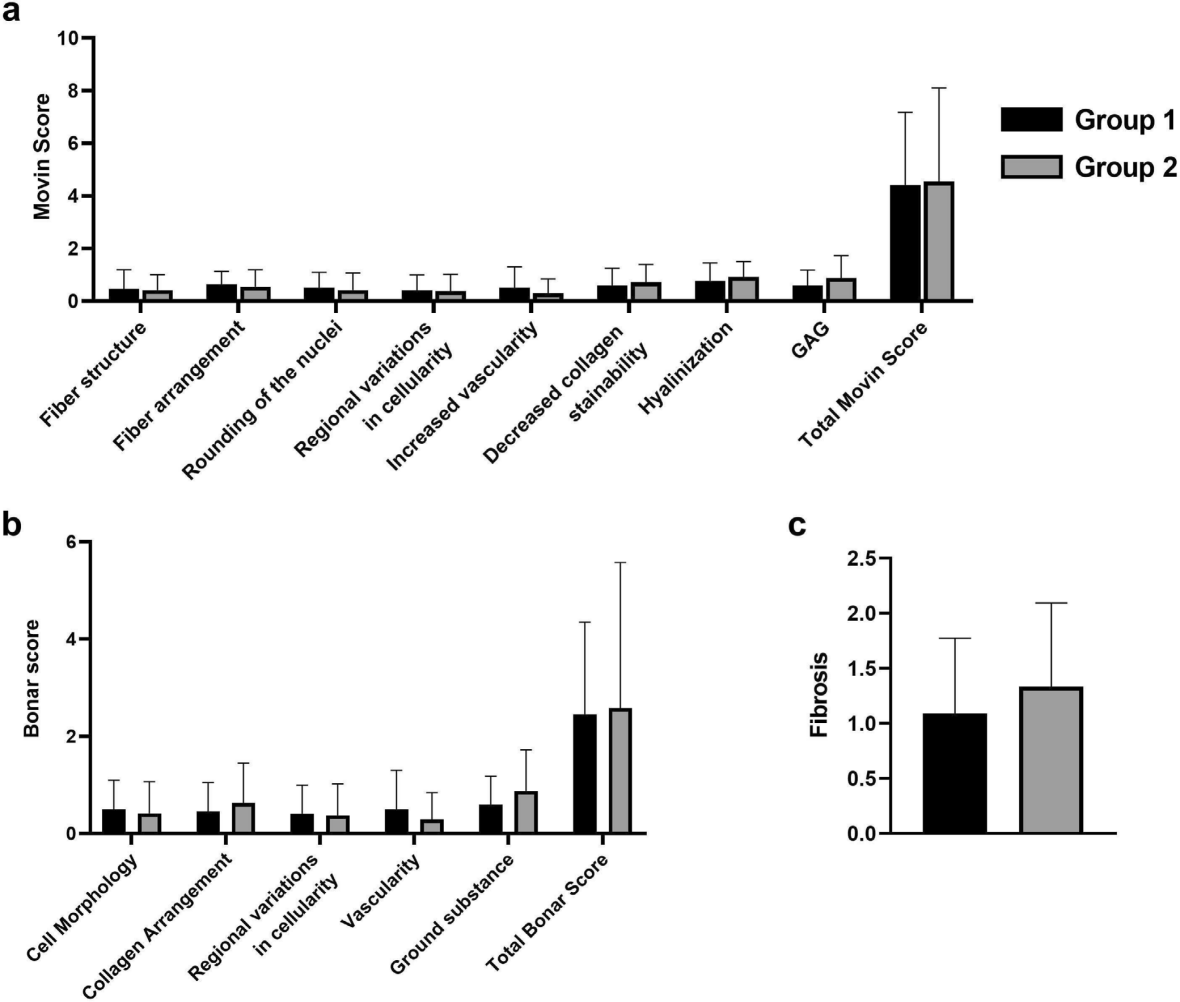
(a) Comparative mean semi‐quantitative Movin scores by group. (b) Comparative mean Bonar scores by group. (c) Comparative mean fibrosis scores by group.

The mean total semi‐quantitative Movin score was 4.7 ± 2.8 in Group 1 and 4.4 ± 3.5 in Group 2 (Figure 2A). The mean values of the parameters included in the semi‐quantitative Movin scoring are shown in Figure 2A, and the distribution of the parameters is presented in Table 2. According to the total semi‐quantitative Movin scores, the result was normal in 3 patients in Group 1, whereas mild degeneration was detected in 16 patients and moderate degeneration in 3. As for Group 2, the result was normal in 3 patients, while there was mild degeneration in 19 patients and moderate degeneration in 3 (Table 3). Based on these total semi‐quantitative Movin scores, patients in Group 2 had more histological changes compared to Group 1, although without a statistically significant difference (*p*:0,96).

**TABLE 2:** Distribution of ACL pathologies by Movin score.

Variable	Group 1 (*n* = 22)				Group 2 (*n* = 25)				*p*
	0	1	2	3	0	1	2	3	
Fiber structure	14	5	3	0	16	8	1	0	0.44
Fiber arrangement	8	14	0	0	13	10	2	0	0.15
Rounding of the nuclei	11	10	1	0	17	6	2	0	0.29
Regional variations in cellularity	13	8	1	0	18	5	2	0	0.43
Increased vascularity	14	5	2	1	18	6	1	0	0.62
Decreased collagen stainability	10	10	2	0	11	11	3	0	0.94
Hyalinization	7	12	3	0	6	16	3	0	0.79
GAG	9	12	1	0	10	10	4	1	0.41

**TABLE 3:** Distribution of ACL pathologies by total semi‐quantitative Movin score.

	Group 1 (*n* = 22)	Group 2 (*n* = 25)
Normal (0)	3 (13.6%)	3 (12%)
Mild (1–8)	16 (72.7%)	19 (76%)
Moderate (9–17)	3 (13.6%)	3 (12%)
Severe (18–24)	0 (%)	0 (%)

The mean total Bonar score was 2.6 ± 1.9 in Group 1 and 2.5 ± 2.9 in Group 2 (Figure 2B). The mean values of the parameters included in Bonar scoring are shown in Figure 2B, with the distribution presented in Table 4. In Group 1, the total Bonar scores showed a normal result in 3 patients, mild degeneration in 15 patients, and moderate degeneration in 4. As for Group 2, 6 patients had a normal result, while 13 had mild degeneration, 5 had moderate degeneration, and 1 patient had severe degeneration (Table 5). Based on these Bonar scores, patients in Group 2 had more histological changes compared to Group 1, although there was no statistically significant difference (*p*:0,55).

**TABLE 4:** Distribution of ACL pathologies by Bonar and fibrosis scores.

Variable		Group 1 (*n* = 22)				Group 2 (*n* = 25)				*p*
		0	1	2	3	0	1	2	3	
Bonar	Cell Morphology	11	10	1	0	17	6	2	0	0.29
	Collagen arrangement	12	9	1	0	15	5	5	0	0.13
	Regional variations in cellularity	13	8	1	0	18	5	2	0	0.43
	Vascularity	14	5	2	1	18	6	1	0	0.62
	Ground substance	9	12	1	0	10	10	4	1	0.41
Fibrosis		4	12	6	0	4	11	9	1	0.69

**TABLE 5:** Distribution of ACL pathologies by total Bonar score.

	Group 1 (*n* = 22) (%)	Group 2 (*n* = 25) (%)
Normal (0)	3 (13.6%)	6 (24%)
Mild (1–4)	15 (68.2%)	13 (52%)
Moderate (5–10)	4 (18.2%)	5 (20%)
Severe (11–15)	0 (0%)	1 (4%)

According to Bonar scoring, 13.6% of Group 1 patients were normal, 68.2% were mild, 18.2% were moderate, and 0% were severe, while 24% of Group 2 patients were normal, 52% were mild, 20% were moderate, and 4% were severe (Table 3).

According to total Movin scoring, 13.6% of Group 1 patients were normal, 72.7% were mild, 13.6% were moderate, and 0% were severe, while 12% of Group 2 patients were normal, 76% were mild, 12% were moderate, and 0% were severe (Table 5).

A comparison of Group 1 and Group 2 in terms of fibrosis (Figure 2C, Table 4) revealed that there were more histological changes in Group 2; however, the difference was not statistically significant (*p*:0,69).

## 4 Discussion

There is only a limited number of histopathological studies in the literature on the degeneration of the anterior cruciate ligament in patients with advanced gonarthrosis [18, 19]. In the present study, we used the Bonar and Movin scoring systems and detected mild‐to‐moderate degeneration in patients with advanced gonarthrosis. We also observed that this degeneration did not increase significantly with the increasing degree of gonarthrosis.

Although aging and trauma are considered the strongest risk factors for osteoarthritis (OA) [20], studies are reporting that the absence of ACL is also an important cause of osteoarthritis [7]. Due to the absence of ACL deficiency as an exclusion criterion in our study, the impact of ACL deficiency on gonarthrosis was not evaluated in this study. A 2021 study conducted in patients with ACL damage whose knees were evaluated with magnetic resonance imaging (MRI) showed rapid progression of cartilage degeneration, especially in the medial tibia, compared to patients with a normal ACL [21]. There are conflicting publications on the reduction of gonarthrosis after reconstruction in patients with ACL rupture [22, 23]. Thomas et al. reported that OA developed in approximately 40% of patients after ACL reconstruction [24]. ACL reconstruction is performed in patients to address their existing instabilities and improve functional scores, allowing them to return to their preinjury activity levels. Patients who underwent ACL reconstruction were not included in this study.

ACL has been evaluated in several studies in patients with gonarthrosis [19]; however, degeneration by stage has not been investigated in patients with advanced gonarthrosis. In the present study, ACL samples were collected from patients with Stage 3 and Stage 4 gonarthrosis during arthroplasty and examined histopathologically. Our findings support the presence of ACL degeneration in patients with Stage 3 and Stage 4 gonarthrosis; however, no significant differences were found between these two groups.

Histological analysis is a reliable method to assess the degree of ACL degeneration [18, 19, 25]. Movin and Bonar scoring systems are commonly used for the analysis of tendon histopathology, providing a semi‐quantitative measure of tissue degeneration [17, 26, 27, 28]. Both are highly correlated and assess similar characteristics [17]. Currently, Movin or Bonar systems are used as repeatable scales [27].

Although there are several scoring systems used for the evaluation of the anterior cruciate ligament, none of them appear to be a routinely used approach [28]. Different scoring systems have been used in different publications [29, 30]. Mullaji et al. in their study, correlation was made by comparing histological findings with radiological findings while examining the cruciate ligaments in knees with arthrosis [30]. They found a significant positive correlation between the radiologic grade of arthritis and the degeneration of the ACL. However, a subjective classification was used to determine the histological findings. Without scoring systems, it may be difficult to classify the findings or grade their severity. Allain et al. also perform macroscopic and histological evaluations, but the lack of a scoring system can lead to subjective interpretations [29]. Especially for histological evaluations, classifying the changes in the ligament structure to a certain degree increases the comparability of different samples and makes the results more objective. Both studies effectively examine the effects of osteoarthritis on ligament tissue, however, without a specific histological scoring system. Scoring systems help to make histological evaluations more systematic and objective. Since these scoring systems lack the sufficient level of objectivity seen in Movin and Bonar scores, there is no specific scoring system to evaluate the ACL. Because the ACL has similar histological features to the tendon, Movin and Bonar scoring systems were preferred in the present study. To the best of our knowledge, this is the first study to use Movin and Bonar scores for the histopathological evaluation of ACL.

In 2001, Allain and colleagues evaluated ligaments by grouping them into four stages [29]. In 2008, Mullaji et al. stratified the anterior and posterior cruciate ligaments into five stages, varying from no change (0) to severe changes (3) [30]. The incidence of cruciate ligament degeneration in osteoarthritic patients was higher in the study by Mullaji et al. compared to the study by Allain et al. [29, 30]. They attributed this finding to the fact that tricompartmental osteoarthritis was more common in the patients included in their study. In the present study, 25 patients had tricompartmental osteoarthritis, while the others had bicompartmental osteoarthritis. The different results may reflect this difference in patients.

When Group 1 patients were graded according to their total Movin scores, mild degeneration was achieved in 72.7% of the patients, while this rate was 76% in Group 2. None of the patients had a score reflecting severe degeneration. In total Bonar scores, mild degeneration was seen in 68.2% of patients in Group 1 and 52% of patients in Group 2. The reason why these two scoring systems yield different percentages is that the criteria and unit ranges evaluated in Movin and Bonar are different.

Some patients undergoing total knee arthroplasty exhibit intact anterior cruciate ligaments [31]. Not all patients with total gonarthrosis demonstrate equally distributed arthrosis in all three compartments; particularly, the lateral compartment may not present arthrosis at the same level. Theoretically, these patients could be adequately treated with a bicompartmental knee arthroplasty, minimizing intraoperative blood loss and preserving cruciate ligaments, thereby maintaining the natural kinematics of the knee [32, 33]. Some authors have advocated for bicompartmental knee replacement over total knee arthroplasty due to its association with reduced blood loss and faster rehabilitation [34]. Hence, bicompartmental surgery can be considered in selected cases [31, 35, 36, 37, 38].

In this case report, since the patient's ACL was healthy, instead of a total knee prosthesis, a three‐compartment unicondylar knee prosthesis was applied, preserving the ACL [39]. As seen in our study, some of our patients who underwent total knee prostheses had healthy ACLs. The tricompartmental arthroplasty applied in this case can be applied as a treatment option for patients with healthy ACLs.

In a 2003 study, only 26% of the patients with osteoarthritis were found to have normal histopathological features of the ACL. However, the control group of that study consisted of bone bank donors, patients with above‐knee amputation, and ACL samples from cadavers [19]. In a study Mont et al. conducted with 174 patients, ACL was found to be intact in 43 patients, worn in 85, and torn in 15, while ACL was absent in 31 knees [25]. In their study, 94 patients had Stage 3 disease, and 49 patients had Stage 4 disease. The study did not report in detail what percent of the patients classified as Stage 4 had ruptured ACL. We believe they obtained different results from ours due to the fact that our study did not include patients with ruptured ACL. In the same publication, it was emphasized that 85% of the cases had histological changes [25]. In the present study, histopathological changes were detected in 86.2% of Stage 3 patients and 88% of Stage 4 patients.

Our study is not without limitations. The limitations include the small sample size, that is, 47 patients included in the study, and the fact that MRI results were not available since MRI scans could not be requested in patients with advanced gonarthrosis owing to the retrospective design. The pre‐ and postoperative clinical examinations of the patients were not included as it was thought that they would not affect the results of this study. Furthermore, Stage 1 and Stage 2 patients could not be included since arthroplasty is not indicated in those groups. Finally, the histopathological examination of the posterior cruciate ligament was not included in our study as the prosthesis implanted during the surgical intervention in patients with gonarthrosis is often one that protects the posterior cruciate ligament.

In this study, patients with Stage 3 and Stage 4 gonarthrosis were evaluated, and their ACL samples were examined histopathologically. According to total Bonar and total Movin scores, the findings have shown that there is no statistically significant increase in ACL degeneration with increasing disease stage in patients with gonarthrosis. Further research is warranted to confirm these findings.

## 5 Conclusion

The present study has shown that, according to histopathological evaluation, there is no increase in ACL degeneration similar to the increase in severe degeneration of cartilage with the increasing stage of gonarthrosis. We believe that our study will contribute to the literature since there is only a limited number of studies that include the histopathological evaluation of ACL in patients with advanced gonarthrosis.

## Author Contributions

Elif Polat performed formal analysis, investigation, data curation, visualization, and contributed to conceptualization, methodology, validation, and writing, reviewing, and editing of the original draft. Burak Günaydin contributed to conceptualization, methodology, validation, supervision, reviewing, and editing of the original draft. Sevil Karabağ contributed to conceptualization, methodology, supervision, writing, reviewing, and editing of the original draft. Nurettin Heybeli contributed to methodology, validation, supervision, writing, reviewing, and editing of the original draft. All authors read and approved the final manuscript.

## Conflicts of Interest

The authors declare no conflicts of interest.

## Data Availability

Research data are not shared.
